# Effect of Omega-3 Fatty Acid on STAMP2 Expression in the Heart and Kidney of 5/6 Nephrectomy Rat Model

**DOI:** 10.3390/md16110398

**Published:** 2018-10-23

**Authors:** Hye Won Lee, Su Mi Lee, Mi Hwa Lee, Young Ki Son, Seong Eun Kim, Won Suk An

**Affiliations:** 1 Department of Internal Medicine, Dong-A University, Busan 49201, Korea; lubhyewon@naver.com (H.W.L.); promise131@hanmail.net (S.M.L.); kidney@dau.ac.kr (Y.K.S.); sekim@dau.ac.kr (S.E.K.); 2 Department of Anatomy and Cell Biology and Mitochondria Hub Regulation Center, Dong-A University, Busan 49315, Korea; hero15p@nate.com

**Keywords:** omega-3 fatty acid, STAMP2, heart, kidney

## Abstract

Six transmembrane protein of prostate 2 (STAMP2) is a critical modulator of inflammation and metabolism in adipose tissue. There are no data on the expression of STAMP2 in chronic kidney disease, which is an inflammatory disease related to metabolic disorders. This study aimed to investigate STAMP2 expression in the kidney and heart in 5/6 nephrectomy (Nx) rats, and the effect of omega-3 fatty acid (FA) on STAMP2 expression. Male Sprague Dawley rats were divided into three groups: sham control (0.9% saline), 5/6 Nx (0.9% saline), and 5/6 Nx treated with omega-3 FA (300 mg per kg per day by gastric gavage). The expression of STAMP2 in the kidney and heart were examined by western blotting. Serum creatinine levels were higher in 5/6 Nx rats than in controls. Compared with sham controls, the expression of IκB, NF-κB, NOX4, SREBP-1, and LXR were upregulated and STAMP2 and phosphorylated-AMPK expression were downregulated in the kidney and heart of 5/6 Nx rats. Omega-3 FA supplementation prevented these changes in biomarkers related to inflammation and metabolic lipid disorders. Omega 3-FA supplementation induced the upregulation of STAMP2 protein in 5/6 Nx rats, which was associated with an attenuation of inflammation- and metabolic disease-related markers.

## 1. Introduction

Persistent inflammation is an important component of chronic kidney disease (CKD) that is related to cardiovascular risk and mortality [1]. Metabolic diseases, such as diabetes and dyslipidemia, also impact CKD, and many studies have shown the bidirectional relationship between metabolic disease and CKD [2]. Still, there are no definite cross-talking mediators including inflammation and metabolism in CKD, which is inflammatory disease related to metabolic disorders.

The six transmembrane protein of prostate (STAMP) protein family, which was initially named six transmembrane epithelial antigens of the prostate, was first reported as a prostate-specific antigen [3]. One of the STAMP family proteins, STAMP2, has three molecular functions, metal ion binding, oxidoreductase activity, and phosphogluconate dehydrogenase activity [4]. In humans, STAMP2 is expressed in several tissues, including the adipose tissue, lung, placenta, heart, liver, and prostate [3,5]. STAMP2, also known as tumor necrosis factor (TNF)-α-induced adipose related protein (TIARP), is induced by TNF-α and acts for anti-inflammation [6,7]. STAMP2 seems to have a central role in the regulation of inflammatory and metabolic signals [8,9]. STAMP2 knockout mice displayed elevated expression of proinflammatory mediators and impairment of insulin sensitivity with lipogenic pathways [7]. In addition, STAMP2 gene overexpression reduced macrophages infiltration and reduced expressions of inflammatory cytokines in epididymal white adipose tissue [10]. Sterol regulatory element-binding protein 1 (SREBP-1) also induces lipid accumulation and increases proinflammatory cytokines [11]. Adenosine monophosphate-activated protein kinase (AMPK) is another sensor for metabolism and inflammation [12,13]. However, it is not clear whether STAMP2 expression is associated with SREBP-1 and AMPK.

Omega-3 fatty acids (FA) have anti-inflammatory and anti-fibrotic effects in the remnant kidney in the 5/6 nephrectomy (Nx) rat model [14], and they show cardioprotective effects in patients with CKD [15,16]. Omega-3 FA was initially used for the treatment of hypertriglyceridemia, and then used for the resolution of renal inflammation, even in rheumatoid arthritis [17,18,19]. Therefore, omega-3 FA may be closely related with inflammation and lipid metabolism. However, there is no report on the effect of omega-3 FA on the expression of STAMP2 especially in CKD.

This study aimed to not only investigate STAMP2 expression in the kidney and heart in 5/6 Nx rats, but also evaluated the effect of marine source omega-3 FA on STAMP2, SREBP-1 and AMPK expression in this model.

## 2. Results

### 2.1. Laboratory Data

The results obtained at 45 days are summarized in Table 1. At the end of the experiment, body weights were significantly lower in rats that underwent 5/6 Nx than in the sham control rats. The body weights of 5/6 Nx rats treated with omega-3 FA were significantly higher than those of the 5/6 Nx rats. Blood urea nitrogen (BUN) and serum creatinine (sCr) levels were higher in the 5/6 Nx and 5/6 Nx-omega-3 FA groups than in the control group. Total cholesterol (TC) and triglyceride (TG) levels were significantly elevated in the 5/6 Nx group, and these levels showed a decreasing tendency after treatment with omega-3 FA.

### 2.2. STAMP2 Data

**Figure 1:**
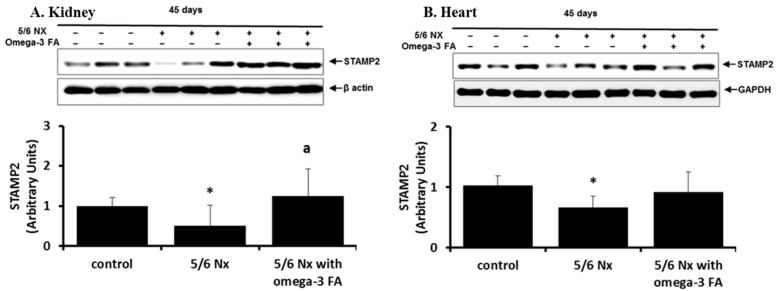
STAMP2 expression in the kidney (**A**) and heart (**B**) in 5/6 nephrectomy rats. Decreased STAMP2 expressions in the kidney (**A**) and heart (**B**) of 5/6 nephrectomy rats were recovered by omega-3 FA. * *p* < 0.05 (mean value is significantly different from the control group). ^a^ *p* < 0.05 (mean value is significantly different from the 5/6 nephrectomy group).

STAMP2 data are shown in Figure 1. Compared with the control group, the 5/6 Nx group showed significant decreases in STAMP2 expression in the kidney and heart (*p* = 0.024 and *p* = 0.024, respectively). Following omega-3 FA supplementation, STAMP2 expression in the kidney was significantly higher, and expression in the heart tended to increase.

**Figure 2:**
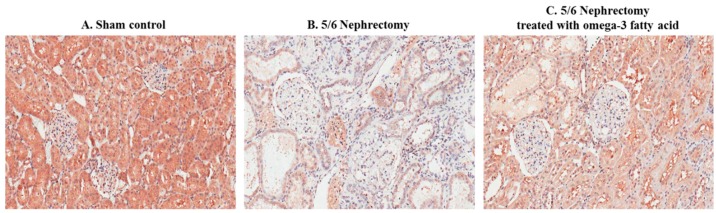
Immunohistochemical staining of STAMP2 in the kidney of sham control rats (**A**), 5/6 nephrectomy rats (**B**), and 5/6 nephrectomy rats treated with omega-3 FA (**C**). (magnification, 200×).

Immunohistochemical staining of STAMP2 in kidney sections is shown in Figure 2. STAMP2 was mainly expressed in the tubules of sham control rats (Figure 2A). In comparison, STAMP2 expression was markedly reduced in the kidney of 5/6 Nx rats (Figure 2B). However, STAMP2 expression in the kidney of 5/6 Nx rats was increased by omega-3 FA supplementation (Figure 2C).

### 2.3. IκB, NF-κB, and NOX4 Data

**Figure 3:**
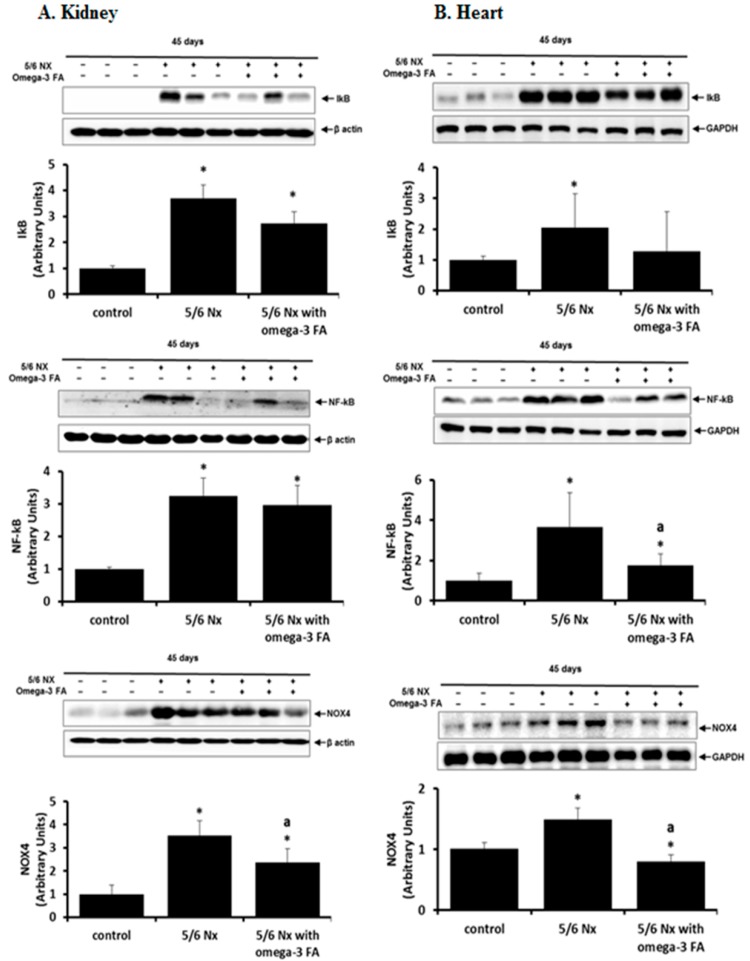
Expression of IκB, NF-κB, and NOX4 in the kidney (**A**) and heart (**B**) in 5/6 nephrectomy rats. IκB, NF-κB, and NOX4 were decreased by omega-3 FA supplementation. * *p* < 0.05 (mean value is significantly different from the control). ^a^ *p* < 0.05 (mean value is significantly different from the 5/6 nephrectomy group).

Compared with the control group, the 5/6 Nx group showed increased inhibitor of kappa B (IκB) and nuclear factor (NF)-κB expression in the kidney and heart (Figure 3). Administration of omega-3 FA prevented these increases in the kidney and heart in 5/6 Nx rat. The anti-inflammatory effects of omega-3 FA were more prominent in the heart than in the remnant kidney.

Nicotinamide adenine dinucleotide phosphate oxidase (NOX) 4 expression was activated in the kidney and heart of 5/6 Nx rats, and administration of omega-3 FA reversed this expression (Figure 3).

### 2.4. AMPK, SREBP-1, and LXR Data

**Figure 4:**
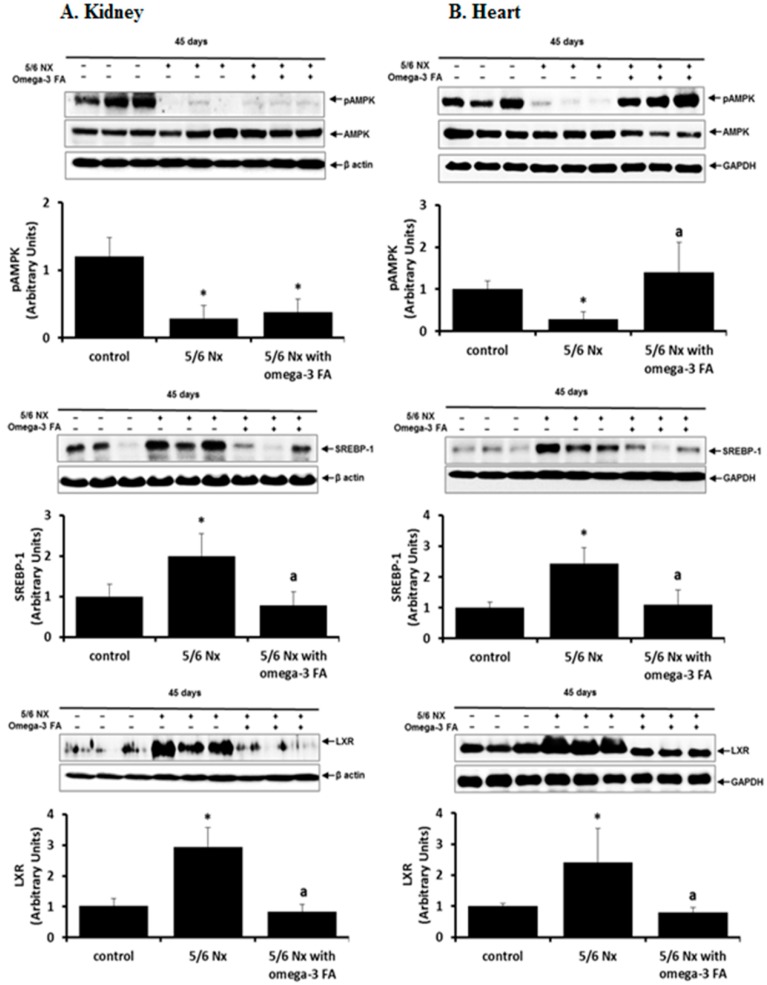
Expression of pAMPK, SREBP-1, and LXR in the kidney (**A**) and heart (**B**) in 5/6 nephrectomy rats. The expression of pAMPK was definitely recovered by administration of omega-3 FA in the heart. * *p* < 0.05 (mean value is significantly different from the control group). ^a^ *p* < 0.05 (mean value is significantly different from the 5/6 nephrectomy group).

Compared with the control group, the 5/6 Nx group exhibited significantly lower levels of phosphorylated-adenosine monophosphate-activated protein kinase (pAMPK) in the kidney and heart (*p* = 0.004, *p* = 0.004, respectively; Figure 4). Omega-3 FA supplementation upregulated pAMPK levels in the heart in 5/6 Nx rats (*p* = 0.004); however, this upregulation was not observed in the remnant kidney.

In the kidney and heart of 5/6 Nx rats, sterol regulatory element-binding protein (SREBP)-1 and liver X receptor (LXR) expression was upregulated compared to the expression levels in the control, and this upregulation was suppressed by omega-3 FA administration.

### 2.5. Renal Pathology

**Figure 5:**
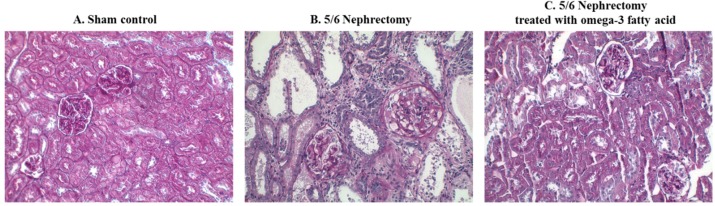
Morphological changes in the kidney of 5/6 nephrectomy rats. Compared to sham control rats (**A**), 5/6 nephrectomy rats (**B**) showed tubular dilatation, tubular atrophy, and interstitial fibrosis. Group treated with omega-3 FA showed less changes (**C**). (magnification, 200×).

The morphological changes observed in the three groups of rats are shown in Figure 5. Compared to the sham control, severe tubular dilatation, tubular atrophy, and interstitial fibrosis were observed in periodic acid-Schiff-stained specimens of the 5/6 Nx group. The 5/6 Nx rats treated with omega-3 FA showed less tubulointerstitial changes in the remnant kidney than the 5/6 Nx rats.

## 3. Discussion

**Figure 6:**
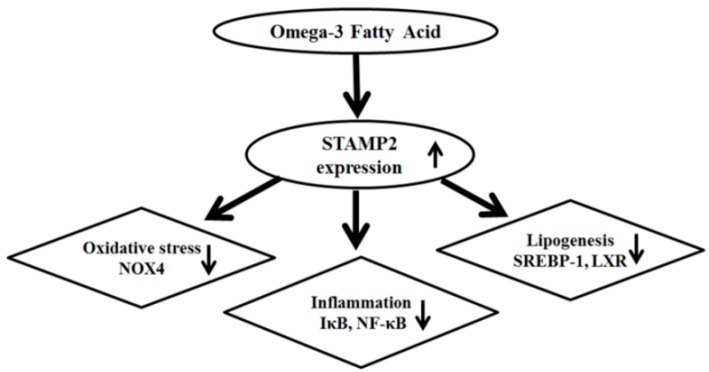
Potential mechanism of omega-3 FA attenuating oxidative stress, inflammation and lipogenesis.

The present study, which is the first to explore the expression of STAMP2 in a CKD rat model, demonstrated attenuated STAMP2 expression in the heart and kidney of 5/6 Nx rats. STAMP2 expression, which was induced by omega-3 FA, may be an important signal for attenuating inflammation, oxidative stress and lipogenesis (Figure 6). A previous study showed that STAMP2 expression in the liver was markedly reduced in non-alcoholic fatty liver disease (NAFLD), which is characterized by lipid accumulation, hepatocellular inflammation, and fibrosis [8]. On the contrary, STAMP2-knockout mice exhibited increased expression of inflammatory mediators such as TNF-α, interleukin 6, and monocyte chemotactic protein 1 [9]. Therefore, we suspect that the decreased STAMP2 levels in the kidney and heart are related to inflammation and fibrosis for kidney function decline and cardiac diseases. Based on present study, we can give a clue that marine source omega-3 FA is helpful for retarding kidney function decline and cardiac disease by STAMP2 up-regulation in CKD. Further studies are necessary to support this assumption.

Under metabolic stress, such as that observed in acute coronary syndrome or diabetes mellitus, oleic acid levels are increased. We previously showed that the levels of oleic acid in erythrocyte membranes were increased in dialysis patients and were decreased by omega-3 FA supplementation [20,21,22]. Overexpression of STAMP2 significantly reduces free FA-induced lipid accumulation, especially in oleic acid-induced NAFLD, by down-regulating adipogenic factors such as SREBP-1. Recent studies have shown that SREBP-1 activation in the kidney induced glomerular sclerosis with up-regulation of pro-fibrotic mediators [23,24]. In this study, omega-3 FA decreased SREBP-1 in the kidney and heart of 5/6 Nx rats, reflecting the anti-fibrotic and anti-adipogenic effect of omega-3 FA. Therefore, increased STAMP2 expression caused by omega-3 FA may be related to beneficial effect for kidney and heart. Lipogenesis is regulated by several transcription factors, including SREBP-1 and LXR [25,26]. STAMP2 gene expression was positively associated with SREBP-1 in human adipose tissue [27]. There is no report of LXR in the study related with STAMP2. In the present study, increased SREBP-1 and LXR were detected in the kidney of 5/6 Nx rats, and up-regulation of STAMP2 induced by omega-3 FA may down-regulate these transcriptional factors. These findings were also observed in the heart of 5/6 Nx rats. Further studies are necessary to clarify signaling pathways of SREBP-1, LXR, and STAMP2.

Adenosine monophosphate-activated protein kinase (AMPK) is a trimeric enzyme consisting of a catalytic α subunit and regulatory β and γ subunits [28]. It has a central role in maintaining intracellular energy homeostasis by functioning as a cellular energy sensor. Phosphorylation of AMPK transforms cells from actively ATP consuming to actively ATP producing [29]. AMPK activity was reduced in renal cells exposed to metabolic stress, such as in high-fat diet-feed mice or adiponectin-knockout mice [30,31]. This study showed that AMPK activity was reduced in association with the increased release of pro-inflammatory factors (NF-κB, IκB) and oxidative stress marker (NOX4) in the 5/6 Nx group. AMPK phosphorylation was significantly recovered in the heart although this change was not found in the remnant kidney by omega-3 FA supplementation. A recent study reported that AMPK affects the regulation of IκB and NF-κB [32]. There may be cross-talk between AMPK and STAMP2 in metabolism and inflammation. It is not clear whether AMPK activation enhances STAMP2 expression. Therefore, further studies are necessary to elucidate the linkage between inflammation including circulating inflammatory mononuclear cells and metabolism.

The renoprotective effect of omega-3 FA in 5/6 Nx rats may be mediated by the anti-inflammatory and lipid metabolism-improving effects of STAMP2 up-regulation. Patients with CKD show higher mortality rates than the general population, and cardiovascular disease (CVD) is the main cause of death [1,33]. CVD is usually initiated by inflammatory processes, and intake of omega-3 FA is associated with reduced rates of atherosclerotic CVD, arrhythmia, and sudden death [34,35,36]. Omega-3 FA has anti-inflammatory effects, and the reduction in oxidative stress may be closely related to increased STMAP2 expression. It is of note that marine source omega-3 FA beneficially works not only for kidney but also for heart.

In this study, STAMP2 suppression in the heart and kidney was observed in 5/6 Nx rats. STAMP2 activation induced by omega-3 FA supplementation may be a potential mechanism for attenuating inflammation and metabolic disorders. In other words, marine source omega-3 FA is a potential therapeutic agent against inflammatory and metabolic disorders via regulation of STAMP2 signaling. Further studies are needed to elucidate the crosstalk between STAMP2 activity and omega-3 FA effects in patients with CKD.

## 4. Materials and Methods

### 4.1. Animals and Experimental Design

Nine-week-old male Sprague-Dawley rats weighing 320–350 g that had been subjected to a 5/6 Nx along with age-matched male sham control Sprague-Dawley rats weighing 350–390 g were obtained from Japan SLC, Inc. (Shizuoka, Japan). They were subjected to 5/6 partial Nx in a two-step surgery. All animal experiments were performed with the approval of the Institutional Animal Care Committee of Dong-A University (DIACUC-14-4) and were conducted in accordance with the Public Health Service Policy on Human Care and Use of Laboratory Animals. Animals were housed in cages under temperature- and light-controlled conditions and were allowed free access to standard diet (DongA One Corporation, Choongnam, Korea) and tap water. The calories of standard diet were provided by protein (24.34%), fat (6.25%) and carbohydrates (69.41%). The composition of nutrients, vitamin and minerals were qualified by Korean Feed Association.

Rats were divided into three groups. Group 1 consisted of sham control rats, and these rats were administered saline (1 mL per kg per day by gastric lavage) for 45 days. Group 2 consisted of 5/6 Nx rats, and they were administered saline (1 mL per kg per day by gastric lavage) for 45 days. Group 3 consisted of 5/6 Nx rats and they were administered omega-3 FA (Omacor, 300 mg per kg per day by gastric gavage) for 45 days. Omacor (Pronova Biocare, Sandefjord, Norway) is marine-originated omega-3 fatty acid. There were 460 mg of eicosapentaenoic acid and 380 mg of docosahexaenoic acid in 1 g of Omacor. The treatment (dose and administration route) of omega-3 FA was decided by previous study [6]. Rats were fed evenly, and body weight was monitored daily. At 15 weeks of age, all animals were sacrificed under diethyl ether anesthesia, and blood samples were collected from the heart. The blood samples were collected in serum separator tubes and EDTA-containing tubes, centrifuged for 10 min at 3000 rpm, and stored at −80 °C until assay.

The concentrations of BUN, sCr, calcium, and phosphorus were determined by an automatic analyzer (Roche, Mannheim, Germany). TC and TG levels were measured using colorimetric test kits (Asan Pharmaceutical Co., Seoul, Korea).

### 4.2. Histopathologic Evaluation

On the day of sacrifice, the kidney and heart were fixed in 10% buffered formalin and embedded in paraffin. Sections (4-μm thick) were stained with periodic acid-Schiff to assess histopathological injury. Kidney lesions, such as tubular atrophy, inflammatory cellular infiltrates, and interstitial fibrosis were evaluated by using an Aperio ScanScope (Aperio Technologies, Vista, CA, USA).

### 4.3. Immunohistochemical Staining

For immunohistochemical staining of STAMP2 in kidney sections, the sections were transferred into 10 mmol/L citrate buffer solution (pH 6.0). Then, the slides were microwaved on medium power for 10 min for antigen retrieval. To block endogenous peroxidase activity, the tissue sections were incubated with 3% H_2_O_2_ in distilled water for 10 min and then with 5% normal goat serum at room temperature for 1 h. Next, the slides were incubated with anti-STAMP2 antibody overnight at 4 °C, and with the secondary antibody for 1 h at 37 °C. Then, slides were incubated in 3,3-diaminobenzidine + H_2_O_2_ substrate and hematoxylin. Negative controls were stained under identical conditions but with buffer solution instead of the primary antibody. Results were viewed using an Aperio ScanScope.

### 4.4. Western Blotting

Kidney and heart tissues were homogenized in lysis buffer containing 300 mM NaCl, 50 mM Tris-Cl, 0.5% Triton X-100, and a protease-inhibitor cocktail (pH 7.6) and incubated at 4 °C for 30 min. After centrifugation at 14,000 rpm for 20 min at 4 °C, the protein concentrations of the lysates were determined with Bradford protein assay reagent (Bio-Rad, Hercules, CA, USA). Equal protein (40 μg) was loaded onto 7.5–15% SDS/PAGE gels and separated by electrophoresis, and then the proteins in the gels were transferred to a nitrocellulose membrane (Amersham Pharmacia Biotech, Piscataway, NJ, USA). The membranes were incubated with each antibody in blocking buffer overnight at 4 °C. The antibodies used included IκB, NF-κB, SREBP-1, NOX4, LXR, and glyceraldehyde-3-phosphate dehydrogenase (GAPDH); all obtained from Santa Cruz Biotechnology (Santa Cruz, CA, USA). Antibodies against AMPK, pAMPK, and STAMP2 were purchased from Cell Signaling Technology (Beverly, MA, USA) and Proteintech (Chicago, IL, USA). Antibodies against β-actin were obtained from Sigma (St. Louis, MO, USA). The membranes were subsequently incubated with horseradish peroxidase-conjugated secondary antibody for 60 min at room temperature. Immunostaining with antibodies was detected with the Super Signal West Pico enhanced chemiluminescence substrate (Thermo Scientific, Hudson, NH, USA) and imaged with an LAS-3000 Plus (Fuji Film, Tokyo, Japan). Quantification and normalization to the β-actin control was conducted using ImageJ 1.48q (National Institutes of Health, Bethesda, MD, USA).

### 4.5. Statistical Analysis

Statistical analyses were performed using SPSS 18.0 software (SPSS Inc., Chicago, IL, USA). Data are presented as mean ± SD or frequency (count and percentage). The characteristics of the study subjects in the three groups were analyzed by using the Mann-Whitney *u* test for continuous variables, the chi-squared test for categorical variables, and the Kruskal-Wallis test for continuous variables. *p* values less than 0.05 were considered statistically significant.

## Figures and Tables

**Table 1:** Characteristics of the rats in each experimental group.

	Control (n = 6)	5/6 Nephrectomy (n = 6)	5/6 Nephrectomy with Omega-3 Fatty Acid (n = 6)	*p* Value
Final Body Weight (g)	463.1 ± 8.6	397.7 ± 21.1 *	429.7 ± 21.3 *^,a^	0.002
Body Weight Gain (g)	91.0 ± 3.0	58.5 ± 22.5 *	82.7 ± 18.6 *	0.006
Blood Urea Nitrogen (mg/dL)	17.7 ± 1.5	77.7 ± 28.4 *	63.9 ± 17.0 *	0.007
Creatinine (mg/dL)	0.4 ± 0.0	1.3 ± 0.6 *	1.0 ± 0.3 *	0.008
Calcium (mg/dL)	6.8 ± 0.3	6.9 ± 0.7	7.2 ± 1.0	0.663
Phosphorus (mg/dL)	8.4 ± 0.4	9.7 ± 4.0	8.2 ± 0.6	0.327
Total Cholesterol (mg/dL)	61.9 ± 7.3	134.2 ± 32.2 *	107.2 ± 37.9 *	0.005
Triglyceride (mg/dL)	134.0 ± 27.2	266.5 ± 87.5 *	216.8 ± 157.4	0.100

Data are expressed as means ± SD. The nonparametric Kruskal-Wallis test was used to compare continuous variables among the three groups. * *p* < 0.05 (mean values are significantly different from the control group). ^a^ *p* < 0.05 (mean values are significantly different from the 5/6 nephrectomy group).
